# Utilization of Ultrashort Bicon Implants in Clinical Situations: A Retrospective Case Series

**DOI:** 10.1055/s-0044-1791786

**Published:** 2024-11-21

**Authors:** Damian Dudek, Gregori M. Kurtzman, Karpe Jacek, Arkadiusz Badziński, Edyta Reichman-Warmusz

**Affiliations:** 1 ArtMedica Outpatient Oral Surgery and Implantology Clinic, Torun, Poland; 2 Academy of Silesia, Katowice, Poland; 3 Implant Cosmetic Dental Center, Silver Spring, Maryland, United States; 4 Department of Neonatal and Pediatric Anesthesiology and Intensive Care, Medical University of Silesia, Katowice, Poland; 5 Silesian Nanomicroscopy Center, Silesia Lab Med-Research and Implementation Centre, Medical University of Silesia, Katowice, Poland

**Keywords:** ultrashort implants, vertical bone loss, insufficient vertical height

## Abstract

When vertical bone loss results in insufficient crestal height to place standard-length implants without the use of osseous grafting, this poses clinical issues to implant usage. Based on an analysis of the literature and clinical experience, it has been found that it is possible to optimally use the available bone volume of the maxillary and mandibular ridges for implant placement without extensive osseous grafting to increase vertical height. This case report will examine several uses of ultrashort implants utilizing the Bicon system in common clinical situations in the maxillary and mandibular arches without the need for osseous reconstruction to improve the available vertical height of the crest to permit implant placement.

## Introduction

Periodontal disease frequently leads to bone and soft tissue loss following extraction of the affected tooth or teeth, which is compounded over time as resorption related to site healing leads to crestal loss of height and in the case of the posterior maxilla, pneumatization of the sinus occurs. 1 Oral reconstructive surgery includes a broad spectrum of procedures to correct that resulting bone loss. Advanced bone loss, especially in the posterior areas of both arches presents challenges to reconstruction with implants and replacement of the missing teeth. In the posterior maxilla, pneumatization of the sinus adds to loss of height crestally yielding a decrease in total available height for implant placement. 2 The posterior mandible has its own anatomical considerations related to the inferior alveolar nerve (IAN), limiting available crestal height which limits implant placement unless extensive grafting is performed to increase ridge height or nerve repositioning is performed. 3 Furthermore, the volume of the anterior maxilla may present some difficulty in using standard-length implants due to the nasal fossa. 4 This can prevent implant placement or require osseous grafting to increase ridge height. Osseous grafting to allow implant placement increases the burden on patients in terms of treatment cost, treatment time, and possible failure of the graft or implants placed within it.

Considering the principle of a patient-centered approach focused on minimizing surgical invasiveness, ultra-short endosseous implants (Bicon) may be an alternative when possible. Those implants are available in 5.0 and 6.0 lengths and have been available for more than 30 years and used in many patients with stable and long-lasting clinical effects being reported. 5 High success rates have been reported for short implants in the posterior and should be considered as a treatment alternative when the alveolar height present is less than 10 mm. 6

The Bicon implant has some unique features compared with other endosseous implants available. The implant's connector does not require a screw to fixate the restorative abutment to the implant, utilizing a Morse (1.5-degree locking) taper connector that when tapped to engage the implant retains the prosthetic part via a frictional fit. This allows the abutment to be rotated circumferentially to position it ideally before the Morse taper is engaged securing the prosthetic part in that orientation. The locking taper creates a bacterial seal avoiding microbial leakage issues that may result in inflammation of the soft tissue-associated peri-implantitis which may lead to bone loss around the implant with the potential of loss of the implant itself. 7 8 9 The implant sloping shoulder versus a flat horizontal shoulder found in most implant designs allows thicker bone at the crestal aspect to aid in its long-term maintenance, allowing the depth of placement options to the surgeon. 10 The plateau or fin design along the body of the implant offers 30% more surface area than threaded implant designs of the same dimensions, allowing mature Haversian bone between the fins of the implant. 11 This cortical-like bone forms at a faster rate of 10 to 50 microns per day in comparison to the appositional bone around nonplateaued implants, which forms at a slower rate of 1 to 3 microns per day. 12 The placement of Bicon implants is essentially a press-fit design at placement due to the horizontal fins versus threads on other implant designs. As a result, the surgical and prosthetic protocols are different from the protocols of most implant systems, only requiring minor modifications in surgical and prosthetic techniques.

The pilot drill operates at a speed of 800 rpm with external irrigation for cooling. Moreover, surgical drills utilized to create the osteotomy may be run at low speed (50 rpm) without irrigation cooling providing increased site control especially when close to anatomical structures such as the maxillary sinus or IAN. As the implant design does not have threads on the body, but fins that are parallel to each other, the implant is not threaded into the osteotomy but pressed into the site at surgical placement. A healing abutment is available in either titanium alloy or polyetheretherketone (PEEK) depending on practitioner's preference. Utilization of the PEEK material has a neutral potentially less irritation effect on the soft tissue. 13 Available prosthetic procedures offer a wide spectrum of solutions for various missing teeth, which are comparable to other implant systems. As the connector between the implant and abutment is a conical Morse taper design, this allows positioning in 360 degrees before engaging the conical connector and securing the abutment. This allows the practitioner more latitude in positioning rotationally than standard connectors found in other implant systems.

## Materials and Methods

The inclusion criteria for implant treatment were as follows:

1. Indications for short implants in the maxilla (distance from the crest to the maxillary sinus <7 mm) and in the mandible (distance from the crest to the IAV canal <7 mm).
2. Confirmation of the volume of bone using the orthopantomogram and cone-beam computed tomography (CBCT) including an alveolar width ≥ 5 mm.

The exclusion criteria were as follows:

1. Lack of written informed consent or willingness to treatment.
2. General contraindications (radiotherapy or chemotherapy, intravenous or oral administration of bisphosphonates) and local contraindications (height of the ridge in the lateral section < 3 mm, width of the process < 5 mm).
3. Exacerbated autoimmune or systemic diseases.

A total of 102 ultrashort Bicon implants were placed in 62 patients from the Kuyavian–Pomeranian Province, Poland. Sixty-two patients (36 females and 26 males) aged 21 to 72 years (mean age of 47.9 years) met the inclusion criteria. The subjects had undergone assessment for reconstructive surgery of the alveolar processes prior to the use of standard endosseous implants. The control group was composed of 60 people (34 females and 26 males) aged 21 to 68 years (mean age of 46.3 years) from the same Province in Poland, in whom, due to good anatomical conditions, 90 standard implants with a standard hexagonal connection were placed with a prosthetic abutment with a diameter of 2.5 mm.

Fifty-seven elective procedures were performed under local anesthesia and five procedures were performed under general anesthesia. Standard site exposure and osteotomy techniques were performed in each case. Soft tissue incisions were made at the top of the alveolar process with the incision line shifted to the palatal side of the maxilla. In 20 cases, closed sinus floor elevation was performed in the lateral sections of the jaws with dedicated instruments. In six patients, sinus abutments were utilized, preventing accidental displacement into the maxillary sinus during healing. Sites were sutured with 5–0 Nylon sutures and removed after 7 days. A 5-month closed healing protocol was adopted to allow for implant integration prior to loading. Next, implants were uncovered, and healing abutments were inserted for 14 days to allow soft tissue healing prior to initiation of the restorative phase of treatment. Finally, prosthetic work was performed. A similar implant-prosthetic treatment was performed in the control group; however, a 3-month closed healing protocol was applied. Details about both groups are given in Table 1 . 14 15

**Table 1:** The study group and control group

Study group		Control group	
**Number of patients**	**62**	**Number of patients**	**60**
Sex F/M	36/26	Sex F/M	34/26
Age (y)/mean age (y)	21–68	Age (y)/mean age (y)	21–68/46.3
Implants in the lateral aspect of maxilla (4–7)	33	Implants in the lateral aspect of maxilla (4–7)	30
Implants in the lateral aspect of maxilla with closed sinus floor elevation + sinus abutment	20 + 6	Implants in the lateral aspect of maxilla with closed sinus floor elevation + sinus abutment	10
Implants in anterior aspect of maxilla (3–3)	2	Implants in anterior aspect of maxilla (3–3)	20
Implants in anterior aspect of mandible (3–3)	0	Implants in anterior aspect of mandible (3–3)	6
Implants in lateral aspect of mandible (4–7)	45	Implants in lateral aspect of mandible (4–7)	24
Implants in regions limited by convergence position of adjacent tooth roots (maxillary/mandibular)	1/1	Implants in regions limited by convergence position of adjacent tooth roots (maxillary/mandibular)	0
Implant diameter and length (mm) 3.5/11.0	2	Implant diameter and length (mm) 3.7/8.0	2
Implant diameter and length (mm) 4.0/5.0	7	Implant diameter and length (mm) 3.7/10.0	32
Implant diameter and length (mm) 4.0/6.0	40	Implant diameter and length (mm) 3.7/11.5	40
Implant diameter and length (mm) 4.0/8.0	13	Implant diameter and length (mm) 4.1/8.0	2
Implant diameter and length (mm) 4.5/5.0	10	Implant diameter and length (mm) 4.1/10.0	10
Implant diameter and length (mm) 4.5/6.0	24	Implant diameter and length (mm) 4.1/11.5	2
Implant diameter and length (mm) 4.5/8.0	2	Implant diameter and length (mm) 4.7/8.0	1
Implant diameter and length (mm) 5.0/5.0	4	Implant diameter and length (mm) 4.7/10.0	1
Total number of implants	102	Total number of implants	90
Osseointegration	97	Osseointegration	84
Lost implants	5	Lost implants	6

## Case Examples

### Posterior Maxilla

**Fig. 1:**
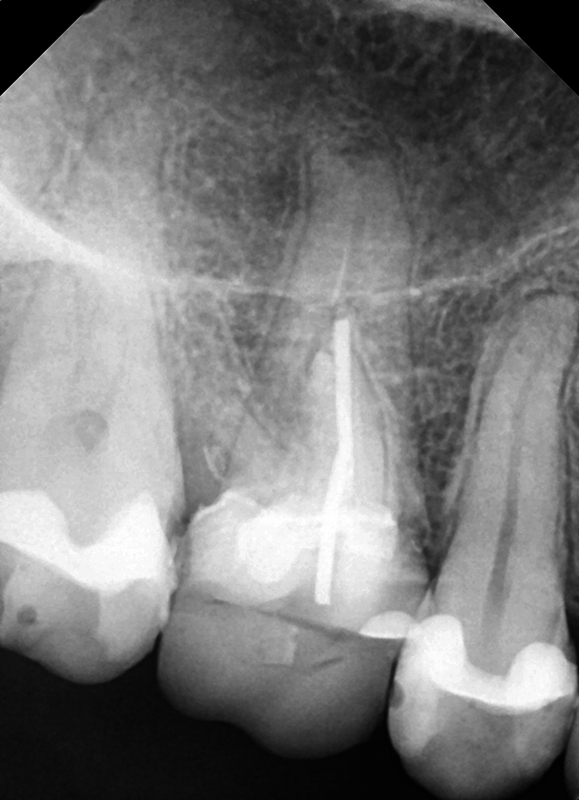
Vertical root fracture identified on the maxillary right molar with enlargement of the maxillary sinus.

**Fig. 2:**
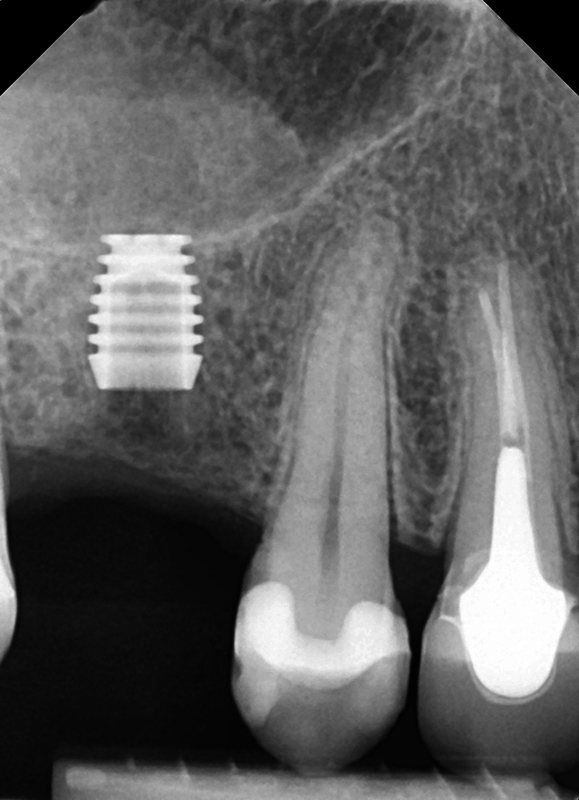
An ultrashort Bicon® implant following placement demonstrating utilization of short available ridge height inferior to the maxillary sinus.

A 40-year-old woman presented with a failing maxillary first molar. Following site healing, an ultrashort Bicon was placed with respect to the maxillary sinus floor ( Fig. 1 ). Restoration was initiated at 4-month postimplant placement and restored with a nonshouldered abutment and crown. The patient was seen on recall and at 12 months, a radiograph was taken to check implant integration demonstrating maintenance of bone associated with the implant ( Fig. 2 ).

### Posterior Mandible

**Fig. 3:**
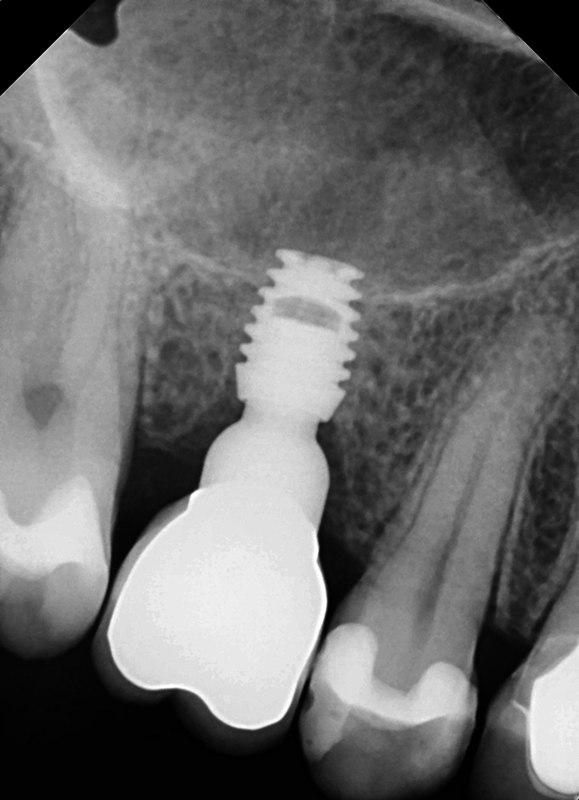
An ultrashort Bicon® implant 12-months post restoration.

**Fig. 4:**
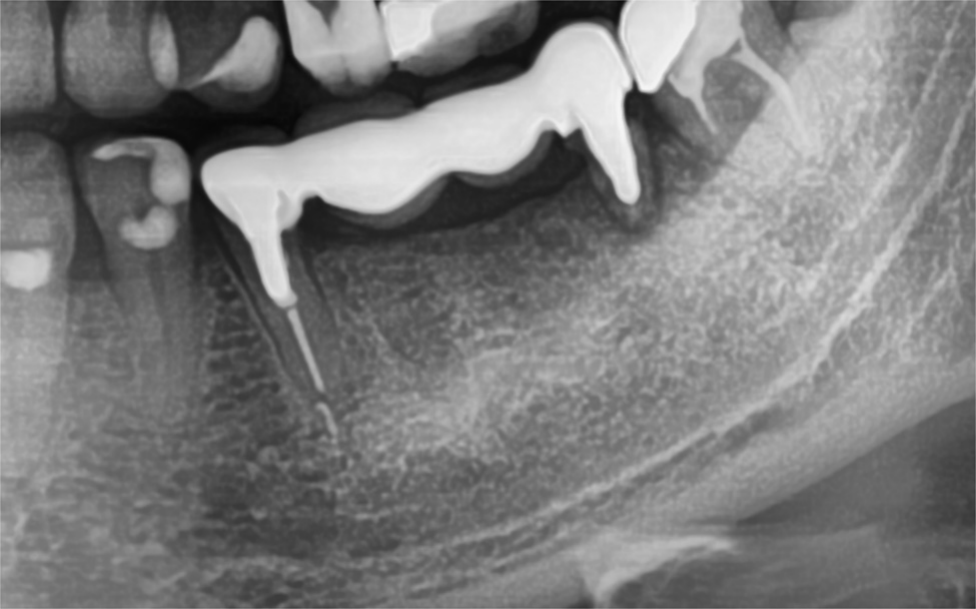
Failing mandibular posterior bridge with mobility and fracture noted on the distal abutment.

**Fig. 5:**
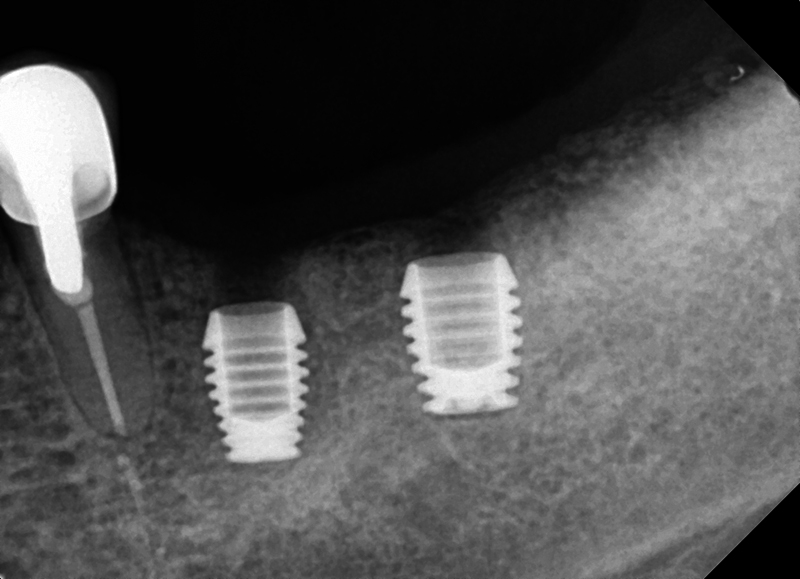
Placement of an ultrashort Bicon® implant to avoid proximity to the inferior alveolar nerve.

**Fig. 6:**
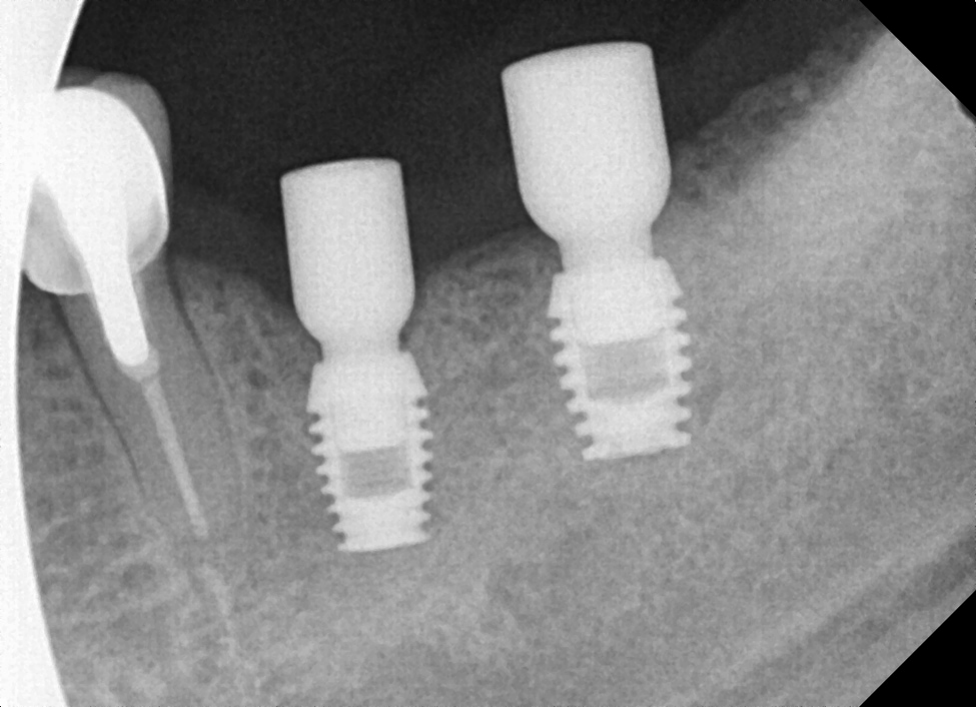
At 4 months post implant placement with radiographic confirmed integration with the surrounding bone, the restorative phase was initiated.

A 39-year-old woman presented with a failing fixed lower left posterior bridge with a bone loss on the distal abutment. The radiograph demonstrated periapical pathology on the distal bridge abutment and in close proximity to the IAN ( Fig. 3 ). The third molar was extracted, the bridge sectioned at the distal of the premolar abutment and the failed second molar was extracted. Ultrashort Bicon implants were placed at the first molar and second premolar sites and allowed to integrate ( Fig. 4 ). At 4 months, postimplant placement initiation of the restorative phase was begun ( Fig. 5 ). The implants were restored with individual restorations. At 2-year recall, a radiograph was taken demonstrating maintenance of crestal bone over that period in function ( Fig. 6 ).

## Results

A survival rate of 95.09% was observed at the 2-year follow-up in the study group and 93.3% in the control group.

Failure of osseointegration in the study group saw the loss of five implants (4.91%). Of those failed implants, all were lost in the posterior mandible. However, in the control group, the lack of osseointegration and loss of six implants (6.7%) after 3 months of closed healing was demonstrated, with five implants lost in the posterior mandible and one in the anterior mandible.

Cortical bone loss in the study group was radiologically confirmed after functional loading in the case of 17 implants (0.17–1.46 mm; mean 0.73 mm) at the 2-year follow-up. No clinical symptoms of inflammation were observed in the study group indicating the crestal bone loss was not related to peri-implantitis. In the control group, atrophy of the cortical bone was noted radiographically with 24 implants at the level of 0.82 to 2.21 mm (mean 0.97 mm). Again, no clinical signs of inflammation were noted in the control group and not likely related to peri-implantitis.

## Discussion

The use of ultrashort implants presented the possibility of the use of minimally invasive implant procedures. Moreover, such procedures reduce the risk of complications, shorten the procedure time to completion of the restorative phase, and reduce treatment costs.

The clinical effectiveness of Bicon implants has been proven. 16 17 18 Maciejewska presented clinical results using ultrashort Bicon implants with the use of these implants in various anatomical conditions where the application of standard implants would have been technically more difficult and with potential complications. 19 They also presented an interesting case in the posterior maxilla, where first they augmented the vertical bone loss with calcium β-triphosphate. Following a period of remodeling and graft organization (8 months), an implant 4 mm × 8 mm was utilized, allowing avoidance of sinus augmentation that might have been further complicated by the patient's chronic sinusitis. 20

Also, the long-term clinical efficacy of ultrashort implants has been widely reported. Lombardo et al compared the use of short and ultrashort implants in the maxillary posterior. 21 They used 93 short and 46 ultrashort implants in 65 patients with different bone volume deficits in the posterior. At the 3-year follow-up, four implants were lost, while implant survival was slightly more favorable for short implants compared with ultrashort implants (97.9 vs. 95.1%).

Furthermore, Marincola considered the use of ultrashort implants to be most effective in some specific clinical situations where necrotic bone loss occurred. 22 This may appear as a result of pathological factors, including mechanical trauma, pathogens, periodontal disease, and coagulation disorders with potentially poorer blood supply, which may lead to microvascular embolism and the formation of defects in the maxillary bones. Marincola reported a case of a patient who presented for implant treatment in whom radiological and clinical assessment confirmed the above-mentioned bone loss in the mandible. Four ultrashort Bicon implants were used (5.0 or 6.0 mm), two in combination with simultaneous augmentation. The implants did not require any fixation and are possible only with the presence of well-preserved cortical bone.

Lombardo et al reported on the long-term clinical efficacy of ultrashort implants. 23 Comparative analysis of the survival rate of single short and ultrashort implants during a 3-year follow-up, where a closed sinus lift procedure was performed simultaneously with implant placement. In a group of 31 patients, 51 ultrashort implants were used: 7 (8 mm), 23 (6 mm), and 21 implants (5 mm) in length, with a success rate reported of 96.08% and mean cortical bone loss was 0.29 mm. An interesting study was presented of a group of 20 patients, where Bicon implants of 6 or 8 mm length and Ankylos implants (Dentsply Sirona, York, Pennsylvania, United States) of 8 mm length were implanted in the posterior mandible with missing molars. 24 Restorations consisted of single crowns at 12-month follow-up including peri-implant clinical parameters (periotest values and per-implant bone changes) with none of the implants lost. Although no significant differences were found in the assessment, the authors indicated better stability of Ankylos implants.

A more extensive study by Urdaneta et al of a 20-month survival analysis of Bicon implants (5.0 × 5.0 mm and 5.0 × 6.0 mm) compared with short implants (5.0 × 8.0 mm) of 410 implants in 291 patients. 18 25 The 211 very short implants and 199 short implants showed similar results with a 97.5% success rate. Nine implants were lost, five measuring 5.0 × 6.0 mm and four measuring 5.0 × 8.0 mm. A slightly higher survival rate was reported for ultrashort (94.6%) compared with short implants (92.2%).

Ultrashort implants have also been used successfully in the treatment of edentulous patients. Ewers performed a study on the implant prosthetic rehabilitation of nine patients with significant bone mass deficits of the maxillary alveolar processes with Bicon implants that were used were 4.0 × 5.0 mm, 4.5 × 6.0 mm, and 5.0 × 6.0 mm in size. 26 Three implants were used in each patient (two in the posterior, while the third implant was inserted centrally into the incisal foramen/nasopalatal canal) with none of the implants lost. They reported the implants were sufficient to stabilize a 12-unit prosthesis based on a glass-fiber reinforced hybrid resin material (TRINIA, Boston, Massachusetts, United States). Furthermore, none of the patients presented posttreatment with the disorders of the nasopalatine nerve.

May et al presented a report on implant placement using the Bicon system in a group of 16 adult acquired immune deficiency syndrome (AIDS) patients (12 males and 4 females) with CD4 <200 cells/μL. 24 All subjects had been undergoing highly active antiretroviral therapy. A total of 33 implants were placed, 3 of which were lost at the 5-year follow-up which consisted of 2 failures of maxillary implants in the anterior and the third in the mandibular posterior. They stressed that AIDS patients could undergo implant treatment. 27 Although the failure rate was 10% in the group compared with widely accepted failure rates in healthy patients at 5 to 7%, the clinical effectiveness of the Bicon system was confirmed in patients with persistent and progressive immune changes at the cellular level.

The presented retrospective study is supported by previous published studies reporting similar results. That data aid in confirming the success of utilizing ultrashort Bicon implants in clinical situations where diminished crestal height would typically require grafting or IAN repositioning to allow the sufficient height to permit implant placement. The success rate presented in the study group parallels implant success rates in general with traditional implant systems.

Restoration of Bicon implants with their Morse taper connector results in a frictional fit between the abutment and implant at the connector. This allows the rotational orientation of the abutment to best align prior to tapping it into the connector to engage the Morse taper. Standard implants utilize nonrotational connectors be they internal or external limiting the rotational orientation of the abutment with the implant. Additionally, as no screw is utilized to fixate the abutment to the implant, screw loosening as reported in standard implant connections is eliminated. This particular implant is designed for delayed loading to allow integration due to its press-fit insertion protocol. The only potential downside to the Bicon implant is in low-density bone or utilization in an extraction site for immediate placement, initial stability may not be achieved.

This retrospective study followed implants placed in private clinical practice over a multiyear period comparing their use of ultrashort implants and standard implants in similar clinical presentations. The clinical results follow data published in other studies, and this retrospective study confirms their results.

## Conclusion

The results of the study presented in the authors' study and the literature show that ultrashort Bicon implants are an important and effective alternative to standard-length implants. This is especially true in those clinical situations where bone volume loss necessitates the use of technically advanced augmentation procedures significantly affecting the patient. Ultrashort Bicon implants should be considered in those clinical situations to decrease complications, treatment time, and cost when supplemental grafting would be required to permit the use of standard-length implants.
